# Attitudes toward the use of ChatGPT to seek oncological information: a qualitative study

**DOI:** 10.3389/fpsyg.2025.1644323

**Published:** 2025-11-25

**Authors:** Ilaria Durosini, Marianna Agnese Masiero, Milija Strika, Davide Mazzoni, Gabriella Pravettoni

**Affiliations:** 1 Department of Oncology and Hemato-Oncology, University of Milan, Milan, Italy; 2 Applied Research Division for Cognitive and Psychological Science, IEO, European Institute of Oncology IRCCS, Milan, Italy

**Keywords:** artificial intelligence, ChatGPT, Psycho-oncology, human-computer interaction, oncology, decision-making

## Abstract

**Introduction:**

The rapid diffusion of new technologies and Artificial Intelligence is revolutionizing access to information for patients with different diagnoses. The spread of ChatGPT, an OpenAI-developed tool that utilizes the GPT language model technology to generate human-like responses based on human questions, also influences how information can be acquired. In this era of technological improvements, it is important to explore people's attitudes toward the use of AI. The main aim of this study is to explore people's attitudes related to using ChatGPT to obtain new information regarding oncological diagnosis through a qualitative data collection.

**Method:**

After reading an ad hoc scenario with an example of a conversation between ChatGPT and a hypothetical user who received an oncological diagnosis, participants were invited to complete online open questions.

**Results:**

A thematic analysis and a Word Association Analysis were conducted on the data collected on 74 Italian participants, revealing both positive and negative emotions. Participants recognized that the use of ChatGPT could help to speed up times and to provide updated information, improving the understanding of complex medical terms. However, concerns about possible negative consequences related to an incorrect use of ChatGPT emerged from the data, in addition to difficulties in understanding the process of data elaboration or data privacy, and the impossibility of replacing the human-doctor relationship.

**Discussion:**

The study underscores the necessity of implementing robust security measures and clear protocols to address concerns and enhance the trustworthiness of ChatGPT in medical contexts, helping the promotion of a safe and correct use of these technologies.

## Introduction

Over the last decades, technological innovations and Artificial Intelligence (AI) have gradually become part of our personal and professional lives. In the healthcare context, software based on machine learning or deep learning is able to analyze a large amount of data related to patients (Durosini et al., 2024; Verlingue et al., 2024; Frasca et al., 2024; Triberti et al., 2021), leading to the improvement of precise clinical diagnosis or personalized treatments (e.g., Ben-David et al., 2024; Masiero et al., 2023). eHealth apps, digital therapeutics, and interactive AIs will also be gradually available for patients with the potential to assist them in their everyday health management. Among the AI technologies which received substantial attention in the last years, there is the Chat Generative Pretrained Transformer (ChatGPT), a chatbot based on a large language model developed by OpenAI. Its barrier-free and user-friendly interface allowed the diffusion and use for multiple applications, including generating textual context. However, the risk of bias and ethical concerns regarding the use of ChatGPT still exists, and further evaluations regarding its applicability in a clinical context are needed. Understanding the attitudes related to the use of ChatGPT for life-threatening diagnoses, such as oncological diagnoses, is of utmost importance. In this line, the main aim of this study is to qualitatively explore the attitudes of potential users related to the use of ChatGPT for obtaining new information regarding an oncological diagnosis and possible future steps. The use of an *ad hoc* scenario with examples of a conversation between ChatGPT and a hypothetical user who received an oncological diagnosis during scheduled screening will help us to assess attitudes related to the application of this kind of artificial entity for retrieving oncological information. Exploring how people perceive conversational AI language models will allow us to identify potential areas for improvement of health-related technologies and potential aspects related to technology acceptance.

## Methods

### Participants

Seventy-four healthy Italian participants were involved in this study (Table 1). Most participants were female (*n* = 45, 60.8%; male: *n* = 29, 39.2%), with an age range between 18 and 72 years old (*M* = 37.16, *SD* = 16.80). Most of them were not in a relationship (70.3%) and had a high level of instruction. Descriptive analyses highlighted that almost half of the participants (*n* = 40; 54.1%) have already used ChatGPT in their lives for different purposes (for work/writing texts: *n* = 33; for personal purposes/fun: *n* = 28; for searching information/for study: *n* = 18), but only 8.1% of them (*n* = 6) use the chatbot for healthcare purposes (e.g., obtaining a description of medical terms related to the interpretation of clinical reports). All the participants took part in this study voluntarily. Data were collected through an online program, Qualtrics, via a non-probabilistic convenience sampling. All participants provided their consent to participate before data collection began.

**Table 1:** Description of participants involved in the study.

	***n***	***%***
**Gender**		
Female	45	60.8
Male	29	39.2
**Education**		
Secondary school	2	2.7
High school	13	17.6
Master degree	39	52.7
Post-degree	20	27.0
**Marital status**		
Single	52	70.3
Married	15	20.3
Divorced	7	9.4
**Occupation**		
White collar	25	33.8
Blue collar	1	1.4
Student/Unemployed	24	32.4
Self-employed	24	32.4

### Procedure

At the beginning of the study, all participants were informed about the functioning of ChatGPT for seeking information within the oncological domain and were invited to read an *ad hoc* scenario with an example of a conversation between ChatGPT and a hypothetical user who received an oncological diagnosis during scheduled screening. The scenario, which differed between males and females, depicted the possible use of ChatGPT as a source of information to better understand the meaning of the scientific language included in a clinical report and possible future steps of care. The screenshots of a potential conversation between ChatGPT and a hypothetical user were also shown to participants in order to increase their understanding of the type and features of the output provided by ChatGPT in response to users' queries.

Taking into consideration the case-scenario, each participant was invited to complete three open questions: 1) What thoughts does using ChatGPT for purposes similar to those presented in the scenario? 2) Do you have concerns about using ChatGPT for purposes similar to those presented in the scenario? 3) Referring to the scenario, what emotions did you experience regarding using ChatGPT for these health purposes?

The open questions were designed to elicit the free expression of participants' attitudes about the use of ChatGPT for retrieval of oncological information, as described in the case-scenario. No time limit or word limit was defined, and participants were invited to describe their thoughts freely.

This study was a part of the project “Validity of Large Language Models (LLM) for the analysis of the experiences of patients with cancer diagnosis”, University of Milan, Italy.

## Data analysis

All text answers provided by participants were in Italian, and the length of the answers ranged from 1 to 154 words. Data analysis followed two different phases. In the first phase, we conducted Word Associations analysis with the software T-LAB Plus 2021 (Lancia, 2021). The tool Word Associations allows to illustrate how co-occurrences relationships determine the local meaning of the selected word (Lancia, 2012; Mazzoni et al., 2018). In our case, T-LAB was used to identify the words that were more frequently used by participants in association (i.e., co-occurring in the same elementary context) with the key term “ChatGPT”. Subsequently, we conducted a thematic analysis to identify principal themes related to using ChatGPT for healthcare purposes. Concerning this, we employed a bottom-up approach, and we did not try to fit the data into pre-existing categories to create a categorization scheme that describes and summarizes the whole data. In particular, Braun and Clarke's (2006, 2021) recommendations for qualitative thematic analysis were used to identify the themes emerging from the data. Two authors (I.D. and M.S.) read the transcripts to understand and familiarize themselves with the qualitative database. An initial coding was performed to identify single segments of the text related to attitudes related to using ChatGPT. The codes were organized into potential main themes and sub-themes. Since some answers expressed more than one concept, that means that each response may include more than one theme or sub-theme. Codes were developed through ongoing interpretative engagement with the data. Following this approach (Braun and Clarke, 2023), codes evolved and changed throughout the coding process, capturing the researcher's deepening understanding of their data. The discussion in repeated meetings allowed the authors to solve any discrepancy and agree on the ongoing process. Lastly, another author (D.M.) reviewed the coding process to further validate the results.

Exemplified quotes that captured concepts of sub-themes were selected from text excerpts. Sixteen responses provided by participants were not coded, as their content was unclassifiable (e.g., “none”, “bad”).

## Results

**Figure 1:**
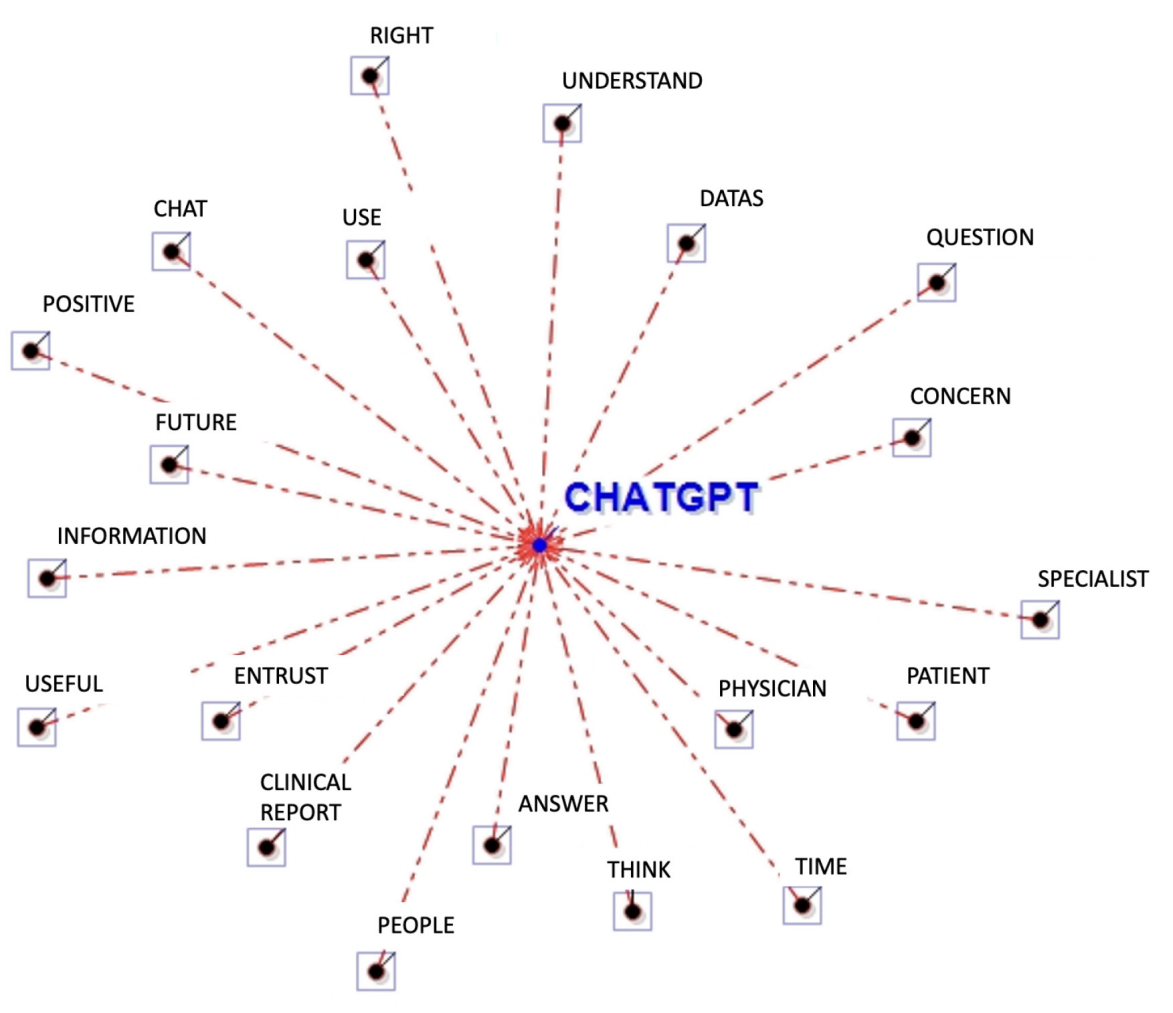
Graphical representation of the word association analysis. In this radial diagram, the lemma ChatGPT is placed in the center. The others are distributed around it, each at distance proportional to its degree of association.

The Word Association Analysis conducted through T-LAB allows one to better understand the attitudes related to using ChatGPT for oncological purposes (see Table 2 and Figure 1). Some words identify the potential user of ChatGPT (i.e., “patient”, “people”) that could be more or less prone to consult a physician or a specialist. Besides the object of interpretation (i.e., “data”), the participants largely used words related to the informative function of ChatGPT and with their own attitudes toward it. In referring to its informative function, participants adopted words like “question” and “answer”, “understand” and “information”. For example, one participant stated, “*I think that ChatGPT could have an informative value”* (ID#14), and another one “*It gave information that was consistent with the question”* (ID#33). Concerning the participants' attitudes toward ChatGPT, participants used words like “positive”, “right”, and “worry”, often accompanied by other words explaining the degree. For example, one participant could use “*a minimum of worry*” (ID#10) or alternatively “*some worries*” (ID#26).

**Table 2:** Results of the word association analysis.

**Lemmas**	**Coeff**.	**EC(B)**	**EC(AB)**	***X* ^2^**	***p***
Physicians	0.516	27	12	6.539	0.011
Response	0.418	14	7	4.620	0.032
Entrust	0.4	5	4	7.629	0.006
Future	0.4	5	4	7.629	0.006
Usage	0.395	8	5	5.723	0.017
Data	0.387	3	3	8.442	0.004
Worry	0.387	12	6	3.832	0.05
Patient	0.353	10	5	3.094	0.079
Think	0.338	7	4	3.555	0.059
Clinical report	0.335	4	3	4.934	0.026

We reported in the table only the first 10 lemmas with the higher coefficients. Coeff, value of the coefficient; EC (B), total amount of elementary context that contains every associated lemma (B); EC (AB), total amount of elementary context where lemmas “A” and “B” are associated (co-occurrences).

Additionally, we conducted a qualitative thematic analysis of all the collected data (Table 3 and Table 4). The themes and sub-themes that emerged from data analysis are presented and discussed in detail below, with sub-themes highlighted in bold.

**Table 3:** Themes and sub-themes extracted using the thematic analysis.

	**Negative aspects**	**Positive aspects**
Emotions (*n* = 60)	- Anxiety-fear-worry (*n* = 25) - Distrust (*n =* 3) - Strangeness (*n =* 7)	- Tranquility (*n =* 1) - Trust (*n =* 21) - Surprise (*n =* 3)
Representations regarding the functioning of ChatGPT (*n* = 19)	Lack of knowledge regarding - the internal working of ChatGPT (black box) (*n =* 13) - Data privacy and—conflict of interest issues (*n =* 2) - Beliefs about the ChatGPT functioning (*n =* 3)	- Access to a huge database of reports (*n =* 1)
Representations regarding consequences of using ChatGPT (*n* = 21)	- Negative impact on health due to incorrect use (*n =* 8)	- Reassure and help patients better understand their condition (clarifying complex medical terms) (*n =* 6) - Informative value (*n =* 3) - Referral to a competent doctor (*n =* 4)
Impact on the relationship between the patient and the doctor/health system (*n* = 19)	Impossibility to replace the relationship with the doctor: - use of ChatGPT without seeking medical advice (like Dr. Google) and lack of human contact (*n* = 13)	- Speed up times (*n* = 6)

Sixteen responses provided by participants were not coded, as their content was unclassifiable.

**Table 4:** Summary of the identified themes and subthemes.

**Themes**	**Description**	**Summary of sub-themes**	**Examples of quotes**
Emotions	Emotions related to the use of ChatGPT to obtain new information regarding oncological diagnosis.	Participants reported both positive and negative emotional responses related to the use of ChatGPT for obtaining health-related information in the oncological field. Negative subthemes encompassed anxiety-fear-worry, distrust (particularly regarding the reliability of the information provided by the chatbot), as well as feelings of strangeness toward ChatGPT. Conversely, positive subthemes included tranquility, trust in ChatGPT, and surprise related to the information provided.	Negative aspects: “*I have fear that ChatGPT is not yet able to give correct answers to my questions”* (ID#59) Positive aspects: “*Using ChatGPT made me feel calm.” (*ID#37)
Representations regarding the functioning of ChatGPT	People's representations of ChatGPT information-processing and functioning.	Participants reported both positive and negative considerations regarding ChatGPT's functioning. Some participants highlighted difficulties understanding the processes behind ChatGPT output or concerns about potential risks associated with data privacy and the risk of dissemination of personal data inserted into AI. Additionally, some participants highlighted the inability to recognize whether the information provided was reliable. On the contrary, the access to a considerable amount of information was perceived as a resource for helping healthcare professionals in their work.	Negative aspects: “*We assume that ChatGPT gives us the right information, but it's impossible to assume it if we don't understand the process”* (ID#45) Positive aspects: ChatGPT “*has great potential. Having access to a huge database of reports, I think that in the future it can help diagnose diseases that perhaps even an expert doctor may miss”* (ID#47)
Representations regarding the consequences of using ChatGPT	People's representation of the consequences of using ChatGPT for health purposes.	Participants reported both positive and negative considerations regarding the consequences of using ChatGPT. The incorrect use of ChatGPT could generate negative thoughts or negative behaviors but also reassure and help patients better understand their condition (e.g., clarifying complex medical terms) or to deepen some aspects (informative value). The referral to a competent doctor also emerged as an important aspect.	Positive aspects: “*Some people could use ChatGPT for a self-diagnosis, without asking for a medical opinion”* (ID#14) Positive aspects: “*ChatGPT can be a good way to understand often complicated medical language”* (ID#73)
Impact on the relationship between the patient and the doctor/health system	ChatGPT's impact on the relationship between patients and healthcare providers.	Participants reported both positive and negative considerations regarding the impact of ChatGPT on the relationship between patients and the doctor/health system. Specifically, participants highlighted the impossibility of replacing the human relationship with the doctor and the related fear of the use of ChatGPT without seeking medical advice, and the lack of human contact. On the other side, participants recognize the potential of ChatGPT to speed up the time, receiving immediate responses to doubts.	*Negative aspects: “Despite ChatGPT suggesting several times to contact a doctor for further clarification, I am afraid that people will rely too much on what is explained with the possibility of misrepresenting the information*” (ID#63) Positive aspects: “*Find answers to some doubts independently”* (ID#74)

### Emotions

The first theme emerged from the data is related to emotions. Participants described that the use of ChatGPT to obtain new information on their clinical conditions could generate both negative emotions and positive emotions. Within the negative area, three different emotions emerged. Twenty-five participants reported anxiety, fear, and worry about the possible use of ChatGPT. Specifically, 10 participants stated feelings of anxiety related to the progression of AI (*Thinking about it makes me feel anxious and want to contact my doctor* ID#13) and nine recognized emotions of fear related to the risk of incurring in incorrect information (“*I have fear that ChatGPT is not yet able to give correct answers to my questions”* ID#59). Six adults reported worry (“*I am worried about the future and the possible use that will be made of AI”* ID#63). In addition, three participants declared distrust, especially related to the reliability of the information provided by the chatbot (“*I feel a little wary because I don't fully know the reliability of ChatGPT”* ID#48). This is related to a perception of insecurity linked to the use of AI, not yet fully known and understood by the population. Additionally, seven participants reported a feeling of strangeness toward ChatGPT. The feeling of strangeness was mainly connected to the uncertainty (*n* = 4) linked to the AI and its functionality (“*I feel uncertain; I wouldn't trust ChatGPT 100%”* ID#9).

On the contrary, participants highlighted positive emotions related to the use of ChatGPT for health purposes. One participants recognized the role of ChatGPT as a “*tool to improve patients' tranquility”* (“*Using ChatGPT made me feel calm”* ID#37) when faced with the numerous negative emotions related to a complex diagnostic picture. Contrary to what was reported by other participants, 21 participants' responses are linked to the possibility of perceiving a sense of trust toward the new artificial entities. Through their different functionalities, AI can convey a sense of security (*n* = 4) and usefulness (*n* = 15) to users. Above all, this sense of security is connected to the possibility of obtaining additional information on questions regarding one's clinical picture. For example, a man stated that using ChatGPT “*could be very useful, especially for those who want to clear up doubts”* (ID#8). The possibility of using ChatGPT for health purposes could, therefore, be positively recognized by users, consequently generating a sense of satisfaction (*n* = 2). Lastly, three participants recognized a sense of positive surprise related to the information provided. In particular, one participant stated “*I was surprised and pleased to see how ChatGPT directs users to consult a doctor*” (ID#31).

### Representations regarding the functioning of ChatGPT

The second theme that emerged from the data is related to people's representations regarding the functioning of ChatGPT. Three negative and one positive sub-themes emerged from the data. The first negative sub-theme is related to the difficulty of understanding the processes behind the ChatGPT output (ChatGPT as a “black box”). Thirteen participants described the ChatGPT elaboration processes as a “black box” and highlighted uncertainty about the appropriate use of the results. The processes that can lead an AI to define a specific response often need to be adequately understood. For example, a man of 27 years recognized that “*We assume that ChatGPT gives us the right information, but it's impossible to assume it if we don't understand the process”* (ID#45). The algorithm that generates the information to date is not yet able to provide an adequate explanation to support the data provided, and the research participants recognized this as a highly negative aspect of using ChatGPT for health purposes. This scenario is related to negative emotions of uncertainty. A woman stated, “*I am afraid I do not understand or misunderstand the information because I do not understand how ChatGPT works”* (ID#66). The second negative sub-theme is related to data privacy. Two participants reported concerns about the possible risks associated with data privacy and the risk of dissemination of personal data inserted into AI. This feeling becomes even greater when ChatGPT is used for health purposes, and when personal information about one's medical condition is inserted into the AI. Therefore, the possibility of not being protected in the privacy of the information included in the AI represents a crucial issue and is still the subject of much attention today. Additionally, one person highlighted the possible conflict of interest with the developers, explaining possible risks related to the secondary purposes of the developers concerning the use of the data “*Concerns about the lack of warnings regarding the use of ChatGPT for similar purposes*” (ID#19). Another negative sub-theme that emerged from the thematic analysis carried out on the responses provided by the participants was the inability to recognize whether the information provided was reliable **(**beliefs about the ChatGPT functioning**)**. Three participants highlighted this aspect as a source of possible negative emotions when using ChatGPT for health purposes. “*Can I use a tool only if I know that information is correct?”* (ID#12): this was underlined by a young man, who recognizes a certain fascination in the use of this AI, but at the same time raises “concern for the possible reliability of the information” (ID#12). These types of thoughts might generate “skepticism” in respondents who expressed the need for “*greater knowledge of the selection criteria for web searches that generate the results”* (ID#44). In particular, these thoughts also tend to be amplified if placed in the context of health, where reading the information can have a strong impact on the user's health decisions and risk-reduction behaviors.

On the contrary, one participant reported one positive sub-theme related to the possibility of using ChatGPT to access a huge database of reports. The possibility of having access to a considerable amount of information was perceived as a resource for helping healthcare professionals in their work. In particular, a man recognized that ChatGPT “*has great potential. Having access to a huge database of reports, I think that in the future it can help diagnose diseases that perhaps even an expert doctor may miss”* (ID#47).

### Representations regarding the consequences of using ChatGPT

The third theme explored is related to the people's representation of the consequences of using ChatGPT. Three negative and three positive aspects emerged from the participants, identifying six sub-themes. The first negative sub-theme is related to the negative impact on health due to an incorrect use of ChatGPT. Eight participants (11%) highlighted the risk that ChatGPT would be used in the wrong way, generating hypochondriacal thoughts and, consequently, inappropriate and unnecessary healthcare decisions. For example, a woman stated that “*Some people could use ChatGPT for a self-diagnosis, without asking for a medical opinion”* (ID#13). Emotions of discomfort may emerge linked to using ChatGPT for health purposes. Some participants recognize the possible “*help of ChatGPT in many areas, but in this area of health, I think it could lead to excessive use and could then tend to make us lose track of reality”* (ID#7). This aspect is particularly relevant, especially considering the specific domain in which the correct understanding of words is key to understanding medical information. ChatGPT's referral to the healthcare professional for further information is certainly considered positive by users. However, others are afraid that this is not sufficient and that it could lead some people to use ChatGPT “*in the wrong way”* (ID#40), “*excessively”* (ID#29), and “*risk losing track of reality”* (ID#7).

On the other hand, using ChatGPT for healthcare purposes could reassure and help patients better understand their condition (e.g., clarifying complex medical terms). Six participants recognize the importance of using ChatGPT to facilitate a better understanding of medical terms. The terms in medical reports can be very complex and difficult to understand by people who are not experts and who “*do not work in the medical field”* (ID#28). ChatGPT can, therefore, be a very useful tool for people to bypass this difficulty and better understand the meaning of some words present in medical reports or presented by healthcare professionals. This aspect is recognized by several participants, including a women who mentioned: “*ChatGPT can be a good way to understand often complicated medical language”* (ID#73). Although many patients' doubts are resolved during the consultation phase, participants recognize that this AI tool, “*perhaps with the help of doctors, could be an excellent tool to relieve and help patients better understand their situation”* (ID#75). Sometimes, ChatGPT can also be a tool capable of managing some situations in which the doctor cannot deal with the patient's doubts and requests. A 20-year-old woman stated, “*it can certainly be a very useful tool, especially for all those cases in which the doctor does not delve deeper and does not explain as he should, lacking empathy”* (ID#5). Consistently, another positive sub-theme is related to the informative value of ChatGPT (*n* = 3). ChatGPT might be used to deepen some aspects, or compare different medical perspectives “*the desire to know more, to make a comparison with what the doctor says”* (ID#64). Besides, the format and the style used to share and present medical information were appreciated by participants. For example, a man aged 21 said, “*I was amazed by the fact that the information was transmitted in some way with empathy and in order not to create alarmism”* (ID#30). ChatGPT can be “*useful for understanding the terminologies but not for the specific case and not for knowing how to proceed”* (ID#14), always referring the final decision to the doctor.

Indeed, the last positive sub-theme that emerged from the data is related to the advice to a specialist. Participants recognized the added value of ChatGPT in the field of health, particularly for its ability to provide information. They were also aware of the importance of the human healthcare professional and encouraged users to consult with a professional (referral to a competent doctor) (*n* = 4). This sometimes generated surprise and happiness among the participants. For example, a man indicated, “*I was surprised and (pleased) with how ChatGPT refers the user to consulting a doctor”* (ID#32).

### Impact on the relationship between the patient and the doctor/health system

The third theme that emerged from the data is related to ChatGPT's impact on the relationship between patients and healthcare providers. Clearly, the data revealed both negative and positive sub-themes related to this specific aspect.

The first negative sub-theme is the use of ChatGPT without seeking medical advice (like Dr. Google) and the lack of human contact (*n* = 13). Participants highlighted the impossibility of replacing the human relationship with the doctor. Some participants expressed fear that ChatGPT could replace human doctors in healthcare practice: “*People rely too much on a computer and less on humans”* (ID#60) and again, “*I am afraid that people rely too much on what is explained with the possibility of misrepresenting the information”* (ID#62). Participants recognized that “*The relationship with the human doctor is irreplaceable”* (ID#42) and the “*Emotional aspect (empathy of the doctor/specialist) in learning or not news related to health”* (ID#23). ChatGPT's recommendation to contact a doctor for further information on the situation may sometimes not be perceived as sufficient by users who recognize that “*Despite ChatGPT suggesting several times to contact a doctor for further clarification, I am afraid that people will rely too much on what is explained with the possibility of misrepresenting the information*” (ID#63), thus raising the risk that “*ChatGPT replaces competent doctors”* (ID#28). This risk seems evident, especially in those people who can use this chatbot “*without the right criteria”* (ID#33), even if this risk is also recognized with other search engines currently used (e.g., “*this risk is equivalent to searching for symptoms on Google without them but ask for medical advice”* ID#33). This situation can also lead people to have a negative attitude toward the doctor: a 20-year-old man raised the concern that using ChatGPT for health purposes could lead to “*the patient arriving at the office already knowing everything and instead not knowing how to contextualize the things written and fully understand them, thus creating a “clash” with the doctor”* (ID#39). The risk, therefore, emerges that AI could be used by people to “*strengthen their position in contrast with the opinion of the treating doctor, strengthened by this acquired “knowledge”, thus hindering the creation of a collaborative relationship with one's doctor”* (ID#73).

One positive sub-theme is related to the potential help of ChatGPT to speed up times (*n* = 6). For example, some participants recognize ChatGPT as a useful aid to “*speed up times related to the world of health”* (ID#2). In line with this, some participants recognized the advantages linked to the “*immediacy of the response”* (ID#59) to obtain rapid and accessible information on one's clinical profile and to “*find answers to some doubts independently”* (ID#74). This can also allow medical professionals to “*avoid receiving further requests from patients following a consultation”* (ID#74). It is, in fact, considered a “*good support tool”* (ID#29) to be integrated—but not replaced—into traditional medical consultation.

## Discussion

The current cross-sectional study explored the specific application of ChatGPT within the healthcare landscape, focusing on its utilization for data interpretation in oncology and its impact on health decisions. We acknowledge that psychological experiences can play a significant role in adopting and accepting ChatGPT in the healthcare setting (Chermack, 2003). In particular, in the field of the cancer care (from prevention to end-of-life), which is characterized by highly preference-sensitive decisions (Masiero et al., 2016, 2019; Lucchiari et al., 2010) involving several affective, cognitive, social and contextual mechanisms shaping the decision-processes. The presence of artificial entities could have an impact on doctor-patient relationship, potentially affecting the decision-making in the health domain (Heudel et al., 2024). AI may also act like a “third wheel” in healthcare and new possible obstacles in the decision-making process could arise (Triberti et al., 2020a). Exploring how people perceive and engage with this form of AI will allow us to identify potential areas for improvement and technology acceptance. By harnessing the model's natural language processing capabilities, we explored how ChatGPT can contribute to the nuanced and complex task of interpreting oncological data, potentially assisting medical professionals in making more informed and timely decisions in the challenging field of cancer diagnosis and treatment (Ferdush et al., 2023). Despite a low percentage of respondents having used ChatGPT for healthcare purposes (8%), the results of the Word Associations revealed a complex and ambivalent representation (rather than negative). This appears consistent with the ongoing process that may lead to a growing number to adopt the chatbot for obtaining health information (Ayre et al., 2025).

The results of the thematic analysis highlighted that ChatGPT and its utilization for health goals might be perceived by the general users as a double face engine having both advantages and disadvantages, that often are strictly interconnected, and their prevalence shapes health decisions. Overall, data retrieved has stressed the informative function of the ChatGPT outcomes and the difficulty of understanding the processes behind the information given by the AI. The majority of the users recognized that ChatGPT boosts the identification of the key health information and provides additional pieces of information increasing comprehension, understanding and being able to “reassure” users. Notwithstanding, several concerns and doubts are related to the sources of such information. Users reported several aspects that might compromise trust and expected utility of the ChatGPT outcomes and contribute to define AI as a “black box”: the reliability of the information provided, uncertainty, data security issues and the risk of dissemination of personal data inserted. This is in line with a critical issue typical of AI software who tend to provide data without detailed information on the process behind the output. One possible solution is the explainable Artificial Intelligence, which aims to improve the ability of an AI to explain the process and the outcomes obtained on the basis of specific input in a more intelligible way (Adadi and Berrada, 2018; Castelvecchi, 2016; Triberti et al., 2020b). Data security and privacy are also significant challenges, as reported by other studies (e.g., Arellano et al., 2018). When it comes to healthcare data, maintaining patient privacy is of paramount importance. ChatGPT requires robust security measures to protect patient information and ensure compliance with privacy regulations. Ensuring secure data transmission, proper encryption, and adherence to privacy guidelines are crucial to enhance confidence in patients and safeguard their personal health information. As ChatGPT interacts with patients and potentially provides medical information, questions may arise regarding who is responsible in case of errors or adverse outcomes. Determining the boundaries of the responsibility and ensuring clear communication about the limitations of ChatGPT are important for mitigating potential legal and ethical concerns.

We argue that issues related to the data privacy, reliability and uncertainty might generate a kind of “rebound effect” related to the user emotional experience associated with the utilization of ChatGPT. Consistently, some users have reported anxiety and fear related to the risk of receiving incorrect or biased information. On the other hand, ChatGPT might have a reassuring function. ChatGPT for healthcare purposes could reassure users, facilitating a better understanding of their clinical situation and of the medical language. Besides, advice received by the ChatGPT, combined with its reassuring function might increase the risk of avoiding a specific medical consultation and to use some self-help strategies to manage their health problems. Additionally, anxiety activation provoked from the ChatGPT outcome might generate hypochondriacal thoughts and, consequently, inappropriate or unnecessary healthcare decisions. This late datum is coherent with the results observed in other studies. For example, Shahsavar and Choudhury (2023), conducted a study to investigate users' intentions regarding the use of ChatGPT for self-diagnosis and health-related purposes. Their findings showed that many users expressed a willingness to use the technology for self-diagnosis. In particular, the study sheds light on how patients perceive ChatGPT's role in addressing health-related questions and self-diagnosis, highlighting the significance of their expectations and decision-making experiences in shaping their intent to use the technology in the healthcare context.

In our study, participants also recognized the importance of maintaining doctor-patient relationship in the healthcare context, considering ChatGPT as a tool to obtain additional information and possibly speed up time, while emphasizing that human contact remains essential in the care pathway. In fact, in the context of oncological care, where negative emotions and uncertainty may affect patients' daily lives, the relationship with healthcare professionals represents an integral aspect of care. Following a cancer diagnosis, patients may have different perceptions of their illness, which can be relatively independent of the clinical characteristics of the disease (Durosini and Pravettoni, 2023). Supporting patients in acknowledging their health conditions, exploring also their personal beliefs, needs, and preferences represent a crucial aspect of the care process (Durosini et al., 2021; Monzani et al., 2021; Sebri et al., 2023). In this context, the active role of psychologists is also relevant for taking care of patients‘ emotions, promoting the curiosity that people have about their own inner world (Aschieri et al., 2020; Aschieri and Durosini, 2015; Aschieri et al., 2016) and promoting patient and caregiver education and information.

Concluding, despite the numerous advantages, unresolved psychosocial considerations are associated with integrating such technologies (Dave et al., 2023; Sebri et al., 2024; Triberti et al., 2021). Understanding the challenges of utilizing ChatGPT for oncological purposes is of utmost importance. The establishment of trust and reliability is one of the most challenging tasks (Prakash and Das, 2020). Patients may be hesitant to fully trust a chatbot for their healthcare needs, especially when it comes to sensitive and critical medical information. Robust validation processes and continuous training using reliable sources are essential. Additionally, accurate control of the information included in the chatbot's output is crucial, as it may occasionally contain “hallucinations” or misleading content.

This cross-sectional study is a first qualitative exploration of people's attitudes toward the use of artificial entities for retrieving oncological information. In this study, participants were asked to read a scenario describing a hypothetical patient consulting ChatGPT to obtain additional information about a clinical report and to better understand the treatment process. While this constitutes an interesting methodological approach, this study was not conducted with cancer patients or survivors. This could represent a potential limitation of this study. Cancer survivors may have increased health knowledge related to cancer compared to the general population due to their previous experience, which may affect how they interpret and use the information retrieved from ChatGPT. Additionally, this study is specifically focused on oncological settings, and therefore the generalization of the findings to other healthcare contexts may not be feasible.

Despite these limitations, the insights emerging from the qualitative open-ended questions may help to promote greater awareness of personal attitudes that can lead individuals undergoing oncological screening or diagnosis to use a generative AI chatbot to seek clinical information. It is essential to raise awareness about the appropriate use of these technologies. At the cultural level, it becomes increasingly important to promote broader awareness of new forms of AI, encouraging a balanced understanding of both potential and limitations, with careful attention to ethical standards and individual privacy. On a practical level, future studies need to further explore people's attitudes related to the use of ChatGPT in the healthcare context in order to orient the refinement of these technologies and the education of the population on their correct use. Helping AI to “speak the human language” and enabling AI systems to describe their processes and outcomes (XAI) could also help to promote a better comprehension of technologies'functioning and outcomes, promoting a more adequate use.

## Conclusion

Technological innovations and AI are increasingly being adopted by individuals for different purposes and across various domains. The use of innovative technologies, such as ChatGPT, within healthcare requires careful consideration to promote their appropriate application and to prevent potential malpractice that could compromise people's health. Encountering life-threatening illnesses, such as receiving an oncological diagnosis, can expose individuals to physical challenges, negative emotions, and changes in their lives. In this study, participants were presented with a scenario describing a hypothetical patient consulting an artificial agent to obtain additional information about a clinical report and to better understand the treatment process. Participants recognized the potential informative value of this tool, helping them to clarify complex medical terms. Participants recognized that the use of ChatGPT could help to speed up times and to provide updated information through access to a huge database of reports, even if the relationship with the human doctor emerged as an essential aspect in the cancer pathway. However, participants highlighted possible concerns related to incorrect use of ChatGPT, including the difficulty in understanding the process of data elaboration, data privacy, and the risk of misrepresenting the provided information. This study highlighted the necessity of implementing robust security measures and clear protocols to address concerns and enhance the reliability and trustworthiness of ChatGPT in medical contexts, helping the promotion of a safe and correct use of these technologies.

## Data Availability

The datasets presented in this article are not readily available because the data that support the findings of this study are available from the corresponding author upon reasonable request. Requests to access the datasets should be directed to Ilaria Durosini, ilaria.durosini@unimi.it.
